# Concurrent Imaging of Markers of Current Flow and Neurophysiological Changes During tDCS

**DOI:** 10.3389/fnins.2020.00374

**Published:** 2020-04-21

**Authors:** Mayank Jog, Kay Jann, Lirong Yan, Yu Huang, Lucas Parra, Katherine Narr, Marom Bikson, Danny J. J. Wang

**Affiliations:** ^1^Laboratory of FMRI Technology, Stevens Neuroimaging and Informatics Institute, University of Southern California, Los Angeles, CA, United States; ^2^Department of Neurology, University of California, Los Angeles, Los Angeles, CA, United States; ^3^Department of Biomedical Engineering, the City College of The City University of New York, New York, NY, United States

**Keywords:** tDCS, dual-echo echo planar imaging (DE-EPI), current mapping, BOLD fMRI, resting-state

## Abstract

Despite being a popular neuromodulation technique, clinical translation of transcranial direct current stimulation (tDCS) is hampered by variable responses observed within treatment cohorts. Addressing this challenge has been difficult due to the lack of an effective means of mapping the neuromodulatory electromagnetic fields together with the brain’s response. In this study, we present a novel imaging technique that provides the capability of concurrently mapping markers of tDCS currents, as well as the brain’s response to tDCS. A dual-echo echo-planar imaging (DE-EPI) sequence is used, wherein the phase of the acquired MRI-signal encodes the tDCS current induced magnetic field, while the magnitude encodes the blood oxygenation level dependent (BOLD) contrast. The proposed technique was first validated in a custom designed phantom. Subsequent test–retest experiments in human participants showed that tDCS-induced magnetic fields can be detected reliably *in vivo*. The concurrently acquired BOLD data revealed large-scale networks characteristic of a brain in resting-state as well as a ‘cathodal’ and an ‘anodal’ resting-state component under each electrode. Moreover, ‘cathodal’s BOLD-signal was observed to significantly decrease with the applied current at the group level in all datasets. With its ability to image markers of electromagnetic cause as well as neurophysiological changes, the proposed technique may provide an effective means to visualize neural engagement in tDCS at the group level. Our technique also contributes to addressing confounding factors in applying BOLD fMRI concurrently with tDCS.

## Introduction

Transcranial Direct Current Stimulation (tDCS) is an emerging neuromodulation technique that has demonstrated therapeutic potential in a range of neurological and psychiatric disorders (Alonso-Alonso et al., 2007; Brunoni et al., 2012; Allman et al., 2016; Kuo et al., 2017; Pontillo et al., 2018), and may improve cognition in healthy subjects (Coffman et al., 2014; Berryhill and Martin, 2018). The advantages of tDCS include its flexibility, simplicity, low-cost, and safety (Bikson et al., 2016). However, clinical translation of tDCS is hampered by the variability of responses observed within and across treatment cohorts (Wiethoff et al., 2014; Li et al., 2015). Addressing this challenge has been difficult due to the lack of an effective means of mapping the neuromodulatory electromagnetic fields together with the brain’s response to the applied stimulation.

To date, attempts to estimate the tDCS current density’s distribution have mostly relied on computational models of the human head (Opitz et al., 2015; Huang et al., 2018). In one case, computational models have shown that although focal current flow distributions can be achieved by employing relatively complex montages (compared to the conventional two-electrode montage), the resultant current-flow patterns are more susceptible to individual differences in anatomy (Mikkonen et al., 2020). Even as these models are validated to a certain degree (Edwards et al., 2013; Huang et al., 2017), clarifying the role of individual anatomy in shaping complex current flow patterns remains a challenge. We recently introduced a novel MRI technique that can measure tDCS current-induced magnetic fields along the static MRI field or Bz (Jog M. V. et al., 2016). The induced fields are direct markers of tDCS currents (by Ampere’s Law), and the technique was able to detect tDCS induced magnetic fields under, and mid-way between the electrodes at the group level. However, it is critical to map these fields in individual subjects as modeling studies suggest that the current distribution depends on the detailed geometry of individual brains (Opitz et al., 2015).

Since our initial work, two additional promising techniques have been proposed. Utilizing Magnetic Resonance Electrical Impedance Tomography (MREIT) (Kasinadhuni et al., 2017; Goksu et al., 2018), these techniques utilize alternating currents to map current-induced magnetic fields (along Bz). The published studies derived current-density distributions under the assumption of zero current density in one direction. In addition, the alternating currents were reported to induce physiological responses rarely experienced with tDCS (e.g., phosphenes and/or heightened sensitivity to pain), and the measurements were limited to a few slices. In addition to the distribution of electric currents, the effects of tDCS are increasingly recognized to be brain network (Pena-Gomez et al., 2012; Kunze et al., 2016) and state dependent (Fox et al., 2014; Li et al., 2019). Consequently, techniques that not only map current-induced magnetic fields across the brain, but also detect the brain’s response to the applied neuromodulation would be a major advance to the field.

A number of studies have employed imaging techniques to monitor brain networks modulated by tDCS, including blood oxygenation level dependent functional magnetic resonance imaging (BOLD-fMRI), arterial spin labeling (ASL), electroencephalography (EEG) and magnetoencephalography (MEG) (Polania et al., 2011; Zheng et al., 2011; Fox et al., 2014; Krishnamurthy et al., 2015; Baeken et al., 2017; Heinrichs-Graham et al., 2017; Donaldson et al., 2019). Collectively these studies suggest that tDCS effects are observed at the network level, are montage (brain current flow pattern) specific, and depend on brain state –at least as imaged prior to tDCS. The precise dependency of tDCS effects on brain networks could be quantified by mapping network dynamics during stimulation.

In this paper we present a technique that, for the first time, provides the capability to *simultaneously* map (i) the tDCS current-induced magnetic fields (a direct marker of the tDCS current), and (ii) BOLD-contrast (a marker of neurophysiological changes) *in vivo*, with full brain coverage.

## Methods

**FIGURE 1:**
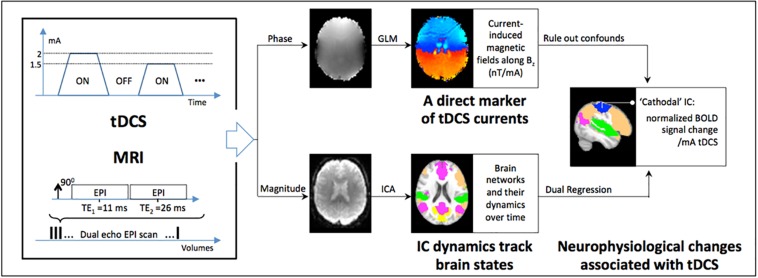
Schematic of the approach for concurrently measuring markers of tDCS currents and the brain’s response *in vivo*: a dual-echo echo-planar imaging (DE-EPI) sequence is applied concurrently with tDCS while the current is varied across blocks of 1.5 min (for details, see text). The phase of the MRI signal encodes the tDCS current induced magnetic fields along Bz (a direct marker of tDCS currents), and is measured by fitting a general linear model (GLM) to the phase data with the applied current as predictor. At the same time, the neurophysiological state of the brain is encoded in the magnitude of the MRI signal as BOLD-contrast. Acquired BOLD data was used in an independent components analysis (ICA) to detect functional networks characteristic of the brain in resting state. Finally, dual regression followed by a 1 sample t-test was used to detect neurophysiological changes in ICA-components underneath the tDCS electrodes (referred to as ‘anodal’ and ‘cathodal’). Additionally, the current induced magnetic field measurements were used to rule out tDCS-induced confounds in the magnitude signal; the latter being sensitive to inhomogeneities in the local magnetic field.

In our proof-of-concept study (Jog M. V. et al., 2016), a standard field mapping sequence was applied for measuring the tDCS current-induced magnetic fields. To improve the number of measurements over time (thus providing increased statistical power) as well as to incorporate BOLD-contrast measurements, a dual-echo echo planar imaging (DE-EPI) sequence was developed. This DE-EPI sequence encodes the induced magnetic fields in the phase, and neurophysiological changes (in the form of BOLD-contrast) in the magnitude of the MRI signal (Figure 1). As a result, the 2^nd^ echo was chosen to be 26 ms, which has previously been shown to be optimal for BOLD-contrast (Jog M. A. et al., 2016). At this TE, the acquired phase data at the 2^nd^ echo accumulates excessive phase wraps, and thus the first echo was chosen to be minimal TE allowed (11 ms), in order to help with phase-unwrapping. The magnitude of the first echo was not used due to inadequate BOLD contrast. The following experiments were performed to validate and evaluate the proposed technique (details of imaging protocol and MRI sequence are described within each experiment).

### Experiments

#### Phantom Validation

**FIGURE 2:**
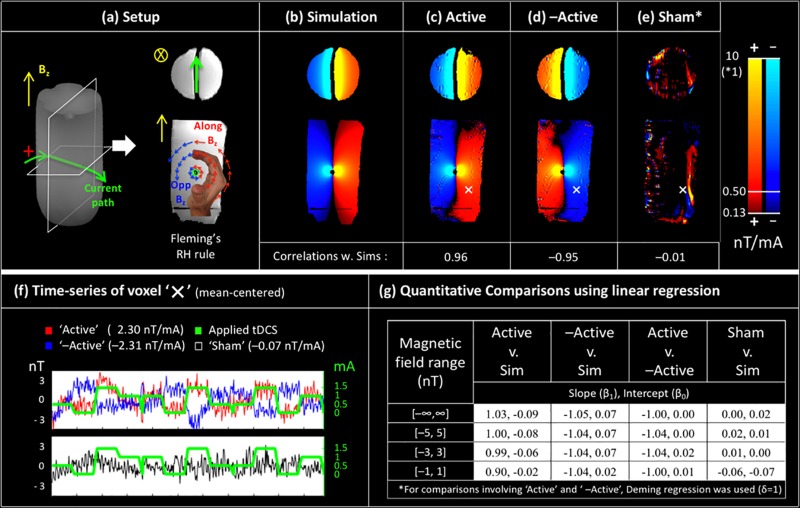
Validation of magnetic field measurements in a controlled environment: **(a)** a custom made phantom with the current induced magnetic fields (along Bz) predicted by Fleming’s right hand rule, **(b)** simulation of current induced magnetic fields using the Biot-Savart law. The experimental measurements from the **(c)** ‘Active’ Session and **(d)** ‘–Active’ Session are in excellent agreement with simulations. Measured fields for the ‘Sham’ session **(e)** are very small (note the different colorscale for ‘Sham’), and spatially uncorrelated with simulations. Times-series of the voxel ‘X’ (shown in **c–e**) are shown in **(f)**. Results of quantitative comparison between measurements and simulations using linear regression are shown in **(g)**. Here, comparisons with simulations indicate accuracy (cols 1–2), between measurements show reliability (col 3), and involving sham show specificity (col 4). Further, these comparisons were performed over progressively weaker ranges of induced magnetic fields (rows) to demonstrate the sensitivity of the field measurements up to 1 nT.

The phantom experiment was designed to validate the accuracy, reliability, sensitivity and specificity of the magnetic field measurements in a controlled environment. Figure 2a shows the phantom design, consisting of a cylindrical bottle of water with an insulated wire running horizontally through it. The insulation confined the electric current to the wire’s path, allowing visualization of the current-induced magnetic fields using Fleming’s right hand rule. Knowledge of the current path also enabled accurate simulations of the current-induced magnetic field (along Bz) for comparison with experimental measurements.

The phantom experiment was performed on a Siemens 3T Trio MRI scanner with a 12-channel head coil. Scan parameters were: TR = 4 s, TE_1_/TE_2_ = 11/26 ms, 90^0^ FA, 3.4 mm × 3.4 mm × 5 mm voxel, 24 slices (ascending), 3004 Hz/Px bandwidth, total scan time = 12 min, 7/8 partial Fourier, GRAPPA acceleration *R* = 2. Second order field shimming was performed. MRI data was acquired from three sessions: ‘Active,’ where the tDCS current was applied; ‘—Active,’ where the direction of the applied current was reversed; and ‘Sham,’ where no current was applied. Each imaging session consisted of three scans, and each scan consisted of 4 different intensities of tDCS currents (0, 0.5, 1, and 1.5 mA) applied in a pseudo-random order with a block design (Supplementary Figure S1A).

#### *In vivo* Evaluations

Two experiments were performed for the *in vivo* evaluation of the proposed method. In Experiment 1, MRI data was acquired from eight healthy participants (4M, mean age: 26 years, ranging from 19 to 49 years) on a Siemens 3T Trio MRI scanner with a 12-channel head coil. Each subject received tDCS with a bilateral montage targeting the motor cortices (Cathode on C3, Anode on C4, 7 cm × 5 cm electrodes). The applied currents were monitored in real-time, and any induced currents in the conductive materials, including electrodes and electrode wires, did not exceed 0.01 mA (0.01 mA being the sensitivity of the ammeter provided in the tDCS-stimulator device). During data acquisition, subjects were asked to relax and fixate on a cross – a typical resting state. The imaging parameters and pulse sequence were the same as the phantom experiment.

In Experiment 2, test–retest data was acquired from seven healthy participants (6M, mean age: 29 years, ranging from 23 to 50 years) using an improved protocol on a Siemens 3T PRISMA scanner with a 20-channel head coil. A faster TR of 2 s was utilized, and each individual tDCS current block’s duration was reduced to 1.5 min in order to improve the sensitivity of BOLD measurements in accordance with (Wang et al., 2003). Remaining scan parameters were: TE_1_/TE_2_ = 11/26 ms, 90^0^ FA, 3.4 mm × 3.4 mm × 4 mm voxel, 35 slices (ascending), 2365 Hz/Px bandwidth, 7/8 partial Fourier, GRAPPA acceleration *R* = 2. Overall, each session (test or retest) consisted of two scans of 12.5 min each, and each scan consisted of four different intensities of tDCS currents (0, 1, 1.5, and 2 mA), applied in a fixed pseudo random order across all subjects (see Supplementary Figure S1B). The same tDCS montage and the resting-state paradigm as Experiment 1 were utilized.

### Data Processing

According to Ampere’s Law, the constant current of tDCS induces a magnetic field that is directly proportional to the applied current (along an orthogonal direction) (Jog M. V. et al., 2016). The current-induced magnetic field can be estimated from the phase of the measured MRI signal:

(1) ϕ=(γ⁢Δ⁢B⁢z⁢T⁢E)⁢m⁢o⁢d⁢2⁢π

where γ is gyromagnetic ratio of protons, *Bz* is the induced magnetic field (along Bz), *TE* is the echo time, and ϕ is the measured phase. The measured phase ranges from 0 to 2π, and measurements outside this range are wrapped back onto it, causing phase-wraps (captured by the modulo operator in the equation).

Since the acquired phase data at the 2^nd^ echo accumulated excessive phase wraps, effective phase unwrapping of the 2^nd^ echo data was achieved using a 4D region-growth algorithm (Barnhill et al., 2015) which utilized phase information from three spatial dimensions as well as temporal dimension (i.e., the first echo, where phase wraps are not as severe). Data from one subject (Experiment 1) was discarded because of excessive phase wraps. Following unwrapping, the 2^nd^ echo phase data was realigned to the first volume using SPM12 (realignment was skipped for the phantom). After linear detrending, the unwrapped phase data was modeled using a general linear model (GLM) with the applied current as the predictor. Nuisance regressors in the model included 6 motion parameters, global signal and regressors accounting for level shifts. The latter were detected between scans, and in regions near the sinus. The slope-estimate of the applied current predictor in the GLM can be interpreted as the magnetic field induced per milliampere tDCS applied.

In parallel, the acquired BOLD-contrast data of the 2^nd^ echo was corrected for motion by realigning all volumes to the first volume, followed by normalization to the MNI space and spatial smoothing using an 8 mm gaussian kernel. All preprocessing steps for the BOLD data were performed using SPM12. Note that the magnitude data of the first echo was not used. To address the possibility of tDCS current induced confounds in the BOLD signal, we modeled the magnitude data from the phantom experiment at the 2^nd^ echo using a general linear model with the applied current as the predictor. Additionally, worst case estimates of the tDCS current induced BOLD confounds were calculated using the magnetic field inhomogeneities measured *in vivo* (see section “Estimation of BOLD Confounds” below).

Finally, a group independent component analysis (ICA) was performed separately on the test and retest datasets using the GIFT toolbox (Calhoun et al., 2009) to identify regions and networks that are consistent across subjects. ICA is a mathematical approach that separates signals into statistically independent sources, and is used in fMRI to identify spatially independent regions that show similar timecourses (i.e., functional brain networks). A gray matter mask was used for the ICA analysis (TPM template in SPM12, 0.25 threshold), and the number of independent components were specified based on the minimum description length (MDL) criterion (Calhoun et al., 2009).

### Statistical Analysis

Measured tDCS current-induced magnetic fields were compared to simulations using the Pearson correlation coefficient. The spatial correlation was performed for all voxels within a mask that included gray matter, white matter and ventricles outputted by SIMNIBS. Additionally, the average measured and simulated field-strengths over the whole brain were compared across subjects, with statistical significance determined using paired *t*-test (two-sided). The repeatability of the measured fields of the test–retest dataset was estimated using the intraclass correlation coefficient (ICC) (Shrout and Fleiss, 1979).

Simulations for the *in vivo* experiments were performed using SIMNIBS (Thielscher et al., 2015). The acquired T1-weighted structural MRI was segmented (using SPM12) to construct a 5-compartment head model in each subject. Default values of tissue conductivities were used (Gabriel et al., 1996; Wagner et al., 2004) and the current-density distribution was simulated. Simulations of the phantom required only a simple threshold based single-compartment segmentation, and no specification of electrical conductivities (because the electric current was confined to the insulated wire). Because of the minimal assumptions involved in simulating the current-induced magnetic field in the phantom, we utilized it as a gold standard to evaluate the proposed technique. The simulated current density distributions were used to calculate the induced magnetic fields using the Biot-Savart Law (as described in Jog M. V. et al., 2016). A quantitative analysis using linear regression was used to verify the accuracy (‘Active,’ ‘—Active,’ vs. simulations), reliability (‘Active’ vs. ‘—Active’), and specificity (‘Sham’ vs. simulation) of the magnetic field measurements. These analyses were performed over different ranges of induced magnetic fields to evaluate the sensitivity of the proposed technique down to 1 nT.

The detected group ICA components were thresholded at *p* < 0.0005 and a minimum cluster size of 1000 to reveal brain networks. The spatial consistency of the detected networks was evaluated using the DICE similarity coefficient (Zou et al., 2004). Additionally, components comprising a singular region underneath each electrode were identified in all datasets, herein referred to as ‘anodal’ and ‘cathodal’ region respectively. Corresponding component timecourses were extracted from these regions for each subject using dual regression, which were subsequently regressed with the applied current waveform. The regression estimates obtained for each subject were used in *t*-test to determine the significance of BOLD-signal changes with the applied current at the group level. All reported *p*-values were two-tailed.

### Estimation of BOLD-Confounds

We additionally investigated the size of potential tDCS current induced confounds in *in vivo* data. Here, magnetic field inhomogeneities resulting from tDCS current-induced magnetic fields were estimated from the gradient of current-induced magnetic fields, and the resulting change in signal magnitude was calculated using the following two models:

(2) $$\begin{array}{cc} f\,S=1-\text{cos}\,\left(\frac{\phi }{2}\right)\,\left[\text{Model}\,\#\,1\right]\; & \\ 1-\frac{\text{sin}\,\left(\frac{\phi }{2}\right)}{\frac{\phi }{2}}\,\left[\text{Model}\,\#\,2\right]\; & \end{array}$$

where *fS* is the fractional signal change, ϕ is the current induced phase changes and determined using Eq. [1]. Model #1 represents the worst-case scenario that assumes maximum field inhomogeneity, while Model #2 assumes a linear spread of field inhomogeneity from 0 to $\left|\vec{\nabla }\,B_{z}\right|$ in a voxel (derivation in Jog, 2017, and shown in Supplementary Material S2). This analysis was performed for the “Cathodal” brain regions where significant changes in the MRI magnitude signal had been observed with the applied current consistently across subjects.

## Results

### Phantom Validation

The phantom experiment was designed to validate the proposed magnetic field measurement technique. Figure 2a shows the phantom, consisting of an insulated current-carrying horizontal wire in a homogeneous medium (water), with the current-induced magnetic fields intuited from the current path using Fleming’s right-hand rule. Quantitative simulations are shown in Figure 2b; note that these are consistent with the predictions from Fleming’s right-hand rule. Figure 2c shows the tDCS current-induced magnetic field (along Bz) as measured during the ‘Active’ session. The measured fields are in almost perfect agreement with simulations (r_Pearson_ = 0.96, *p* < 0.001). In the ‘—Active’ session, the direction of applied currents was reversed, which should result in a sign-flip of the induced magnetic fields. Figure 2d shows the measurements from the ‘—Active’ session, which demonstrates this behavior with the expected sign-flip and an otherwise excellent match with simulations (Figure 2d, r_Pearson_ = −0.95, *p* < 0.001). Finally, Figure 2e shows measurements from the ‘Sham’ session. Here, data were acquired with the stimulator switched off, and we expected to measure zero-mean noise that was spatially uncorrelated with simulations. Measurements were observed to be consistent with expectations, with most of the measured fields below 0.5 nT, and a near zero correlation with simulations (Figure 2e, r_Pearson_ = −0.01). Figure 2f illustrates the magnetic field measurements for each session in a sample voxel marked as ‘X’ over time.

A quantitative comparison between experimental measurements and simulations was performed using a linear regression model. As shown in Figure 2g, slope and intercept parameters were estimated and overall, a statistically significant linear relationship was observed between ‘Active,’ ‘—Active,’ and ‘Simulations,’ with absolute slopes close to 1 and intercepts close to zero [slope, intercept = (1.03, −0.09)_Active, Sim_; (−1.05, 0.07)_–Active, Sim_; (−1.00, 0.00)_Active, –Active_]. These relationships held even when the analysis was performed over progressively smaller ranges of induced magnetic fields down to 1 nT (4^th^ row in Figure 2g), demonstrating the sensitivity of our method. Lastly, ‘Sham’ measurements showed a practically negligible linear relationship with simulations [slope, intercept = (0.00, 0.02)].

**FIGURE 3:**
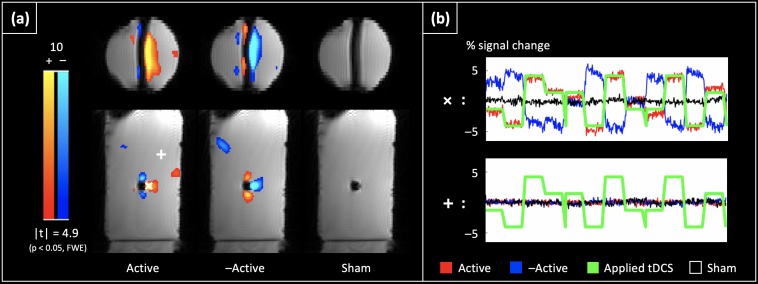
tDCS current induced BOLD confounds in a phantom: **(a)** shows the significant tDCS current induced changes in the magnitude signal at the 2^nd^ echo (*p* < 0.05, Family-wise error corrected). The largest significant changes are observed near the wire, and show a sign change with a flip in the direction of the applied current (compare ‘Active’ vs. ‘–Active’). No such changes are observed with the ‘Sham’ dataset. **(b)** Shows the timecourses from two representative voxels: ‘ + ’ (located near the wire where the largest changes were observed), and ‘ + ’ (located in the bulk). While the largest changes were < 5%/mA near the wire, the timecourses of voxels in the bulk are flat, and uncorrelated with the applied current.

Figure 3a shows the detected significant tDCS current induced changes in the magnitude signal in the phantom (*p* < 0.05, Family-wise error corrected). As can be seen, the largest significant changes are observed near the wire, and the sign of these changes flips with the direction of the applied current (‘Active’ vs. ‘–Active’ cases). No such changes were observed in the ‘Sham’ case. Figure 3b shows the timecourses for the tDCS current induced magnitude changes in two sample voxels: ‘X’ (near the wire), and ‘ + ’ (present in the bulk). The largest changes were < 5%/mA of applied current, and were within 1.5 cm of the wire. There are a few “activated” clusters away from the wires probably due to vibration related signal changes in water.

### *In vivo* Evaluation

#### tDCS Induced Magnetic Field Changes (Along B_*z*_)

**FIGURE 4:**
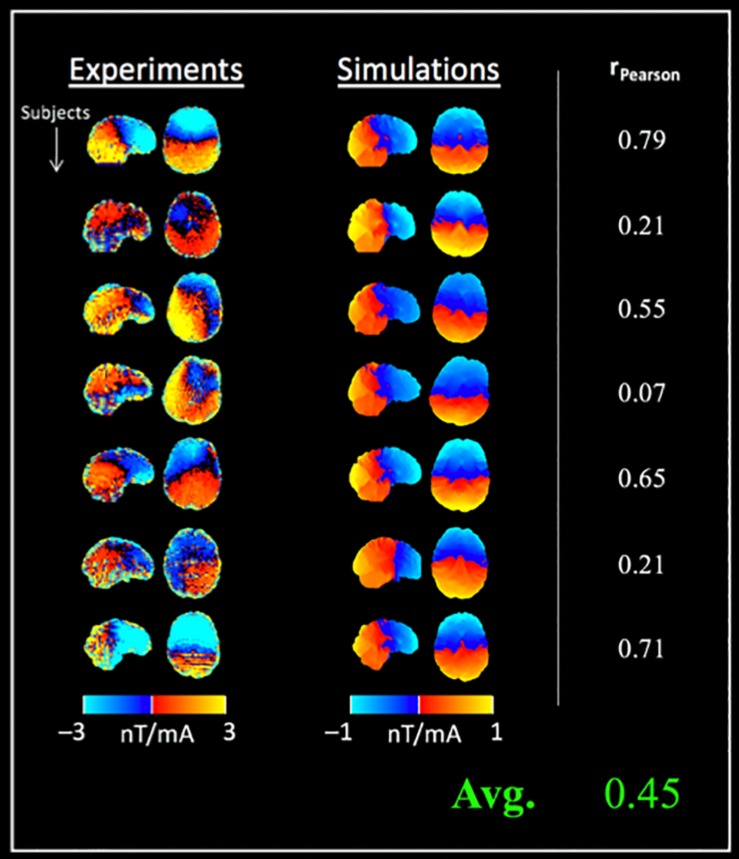
Comparison between tDCS current induced magnetic fields (along Bz) and simulations in a separate cohort: column 1 shows the measured fields, followed by simulation results for seven subjects (rows). Data from two subjects was excluded due to wrap artifacts detected in preprocessing (4D phase-unwrapping step). The *in vivo* measurements and simulations were correlated (r_Pearson_ = 0.45 ± 0.28 on average), and simulations were significantly lower than the measured fields (note the different color scales used for displaying simulated fields).

Figure 4 shows the measured current-induced magnetic fields (along Bz) together with simulations from Experiment 1. The measurements were found to correlate with simulations [average r_Pearson_ = 0.45 (± 0.28), Figure 4], however, the simulated fields were observed to be significantly weaker than *in vivo* measurements (mean difference between simulations and measurements = −1.49 nT, *p* = 0.0048).

**FIGURE 5:**
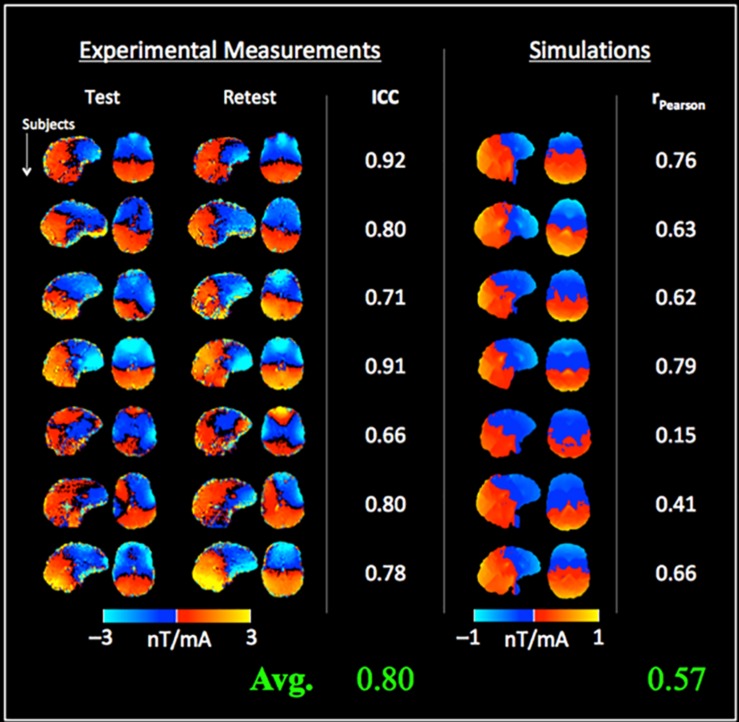
Test–retest measurements of the tDCS current-induced magnetic fields and comparison with simulations: the left columns show the magnetic fields measured from the test and retest sessions, and the intra-class correlation coefficient (ICC) for each subject (= 0.80 ± 0.10 on average across subjects). Simulated magnetic fields are shown in right columns. Although a good correlation was observed (r_Pearson_ = 0.57 ± 0.22), simulations were fractionally lower than the measured fields (note the different color scales used for displaying simulated fields).

Figure 5 shows the measured tDCS current-induced magnetic fields from Experiment 2 with the test and retest sessions. A high mean ICC of 0.80 (± 0.10) was observed between repeated measurements across subjects. Compared to the findings of Experiment 1, the correlation between measurements and simulations was increased [average r_Pearson_ = 0.57 (± 0.22), Figure 5], and the simulated fields were significantly weaker than the measured fields [mean difference between simulations and measurements = −0.9 nT (test), −1.0 nT (retest), *p* < 0.001 in both cases]. In contrast, a non-significant difference of −0.1 nT was observed between test and retest measurements. The systematic difference between simulations and measurements was also indicated by the different color scales required for displaying simulated and measured fields in Figures 4, 5 respectively.

#### BOLD-Contrast Measurements

**FIGURE 6:**
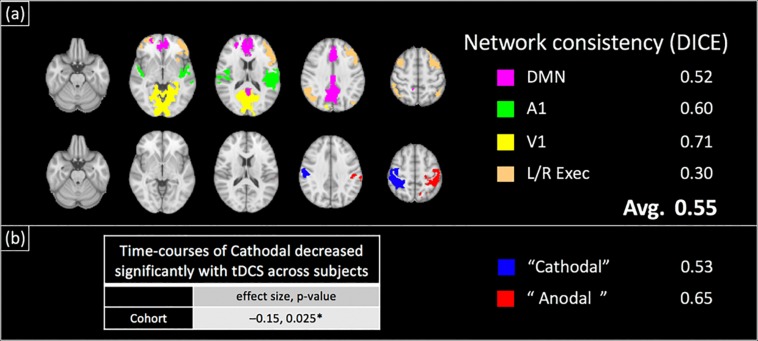
Group-ICA Analysis of BOLD-contrast data acquired concurrently with field measurements in a separate cohort: group ICA revealed large scale brain networks as well as two lateralized motor cortex components (shown in **a,b** respectively). Similar to the test–retest results (Figure 7), the locations of the motor cortex components were verified to be directly underneath the anode and cathode electrodes (Supplementary Figure S2). These components and networks were found to be spatially consistent with their test–retest counterparts (DICE coefficient = 0.55 on average). The time-course of ‘Cathodal’ was found to significantly decrease with the applied tDCS current at the group level ({normalized signal change/mA tDCS, *p*-value} = {–0.15, 0.025}).

Figure 6 shows the results of the group ICA analysis performed on the concurrently acquired BOLD-data. Figure 6a shows the typical resting state brain networks (RSNs) detected, including the primary visual, primary auditory, default mode and executive control networks. The detected networks were consistent with the RSNs of Experiment 2 (see below), with an average DICE coefficient of 0.55 (± 0.14). Additionally, two components, each comprising the lateralized motor cortex, were observed (Figure 6b). Peak coordinates for these components were confirmed to be directly underneath the 7 cm × 5 cm anode and cathode electrodes (see Supplementary Material S3), herein referred to as ‘Anodal’ and ‘Cathodal’ regions respectively. Although no significant changes with applied current across subjects were observed for the ‘Anodal’ region, the normalized BOLD-signal of the ‘Cathodal’ region was observed to significantly decrease with the applied tDCS current at the group level (Figure 6b, normalized signal change/mA tDCS = −0.15, *p* = 0.025).

**FIGURE 7:**
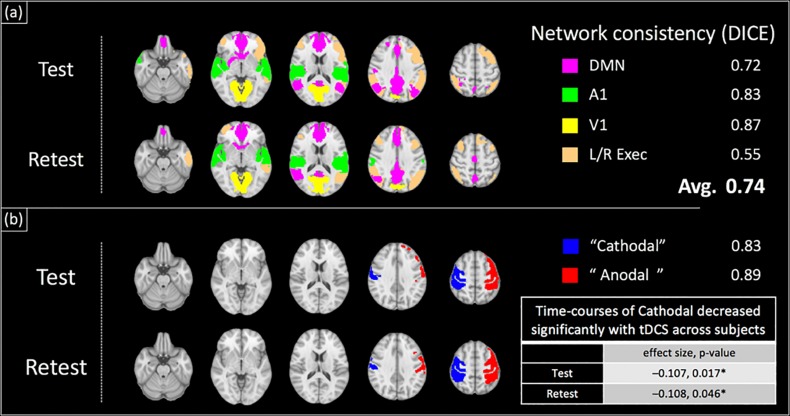
Group-ICA Analysis of BOLD-contrast data acquired concurrently with field measurements (test–retest dataset): a group ICA revealed large scale brain networks as well as components comprising lateralized motor cortex components (shown in **a,b** respectively). The locations of the two motor cortex components were verified to be directly underneath the anode and cathode electrodes placed on C4 and C3 respectively (Supplementary Figure S2). These components and networks were spatially consistent between test and retest sessions, with an average DICE coefficient of 0.74. The time-course of ‘Cathodal’ was found to significantly decrease with the applied tDCS current at the group level ({normalized signal change/mA tDCS, *p*-value} = {–0.11, 0.017}_test_, {–0.11, 0.046}_retest_).

Figure 7 shows the results of the ICA analysis performed on the BOLD-data from Experiment 2 with the test–retest dataset. Similar to Experiment 1, typical RSNs were detected including the primary visual, primary auditory, default mode and executive control networks. The detected RSNs were spatially consistent between test and retest sessions, with an average DICE coefficient of 0.74 (± 0.13). Additionally, two components, each comprising a lateralized motor cortex, were also observed (Figure 7b) and the peak coordinates for the two components were confirmed to be directly underneath the anode and cathode electrodes respectively (see Supplementary Material S3). Similar to Experiment 1, no significant changes with applied current across subjects were observed for the ‘Anodal’ region, while the normalized BOLD-signal of the ‘Cathodal’ region was observed to significantly decrease with the applied tDCS current at the group level [Figure 7b, normalized signal change/mA tDCS, *p* = (−0.11, 0.017)_test_, (−0.11, *p* = 0.046)_retest_].

#### Estimation of BOLD Confounds

**FIGURE 8:**
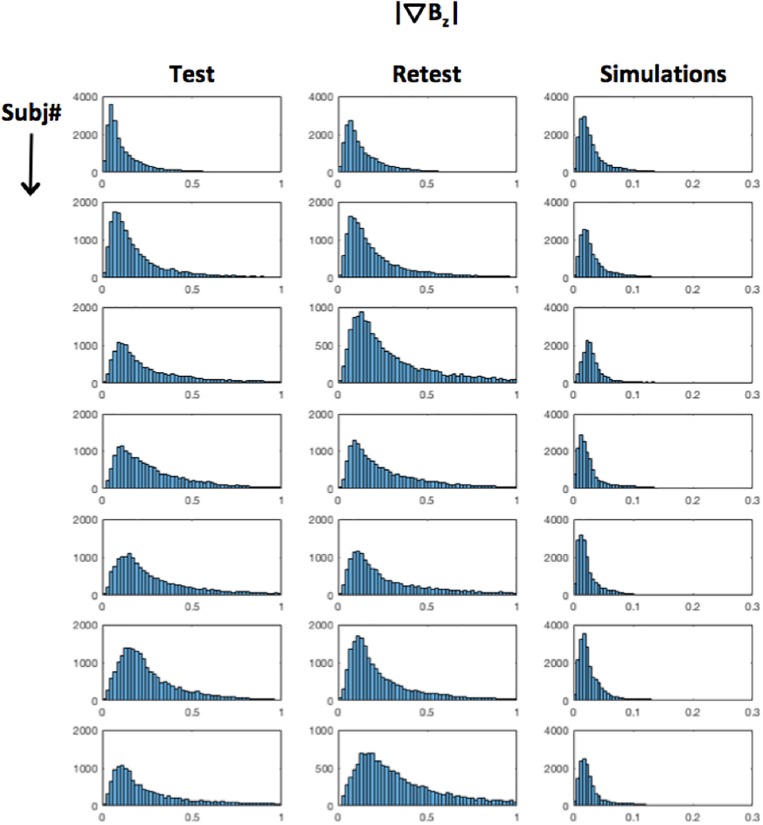
Distribution of $\left|\vec{\nabla }\,B_{z}\right|$ : $\left|\vec{\nabla }\,B_{z}\right|$ histograms for each subject (rows) are shown for test–retest measurements (Columns 1,2) as well as simulations (Column 3). As can be seen, $\left|\vec{\nabla }\,B_{z}\right|$ = 1 nT in the worst case.

Figure 8 shows a histogram of the tDCS induced field inhomogeneity in nanotesla (nT) for each subject (rows), plotted for the test, retest and Simulations (Columns 1–3) respectively in the ‘Cathodal’ region. Even assuming $\left|\vec{\nabla }\,B_{z}\right|$ = ∼1 nT in the worst-case scenario, induced confounds are estimated to be at least an order of magnitude smaller than the experimentally observed effects (Table 1).

**TABLE 1:** Estimated tDCS confounds in the BOLD signal (relative to observed signal changes): worst case $\left|\vec{\nabla }\,B_{z}\right|$ values were used to estimate the tDCS induced confounds in the BOLD signal for the ‘Cathodal’ region (see section “Methods”).

Datasets	Observed-signal-change/mA	| Confound-driven-signal-change/observed|	
		Model #1	Model #2
Experiment 1	−0.15	4.0e-5	1.3e-5
Experiment 2 (test)	−0.11	5.5e-5	1.8e-5
Experiment 2 (retest)	−0.11	5.5e-5	1.8e-5

*Column 1 shows the experimentally observed BOLD-signal change/mA tDCS in the ‘Cathodal’ region, and Cols 2,3 show the worst-case confounds relative to the observed changes. The latter were observed to be at least an order of magnitude smaller than measurements.*

## Discussion

### Magnetic Field Mapping

The key innovation of the proposed technique is the capability to simultaneously image markers of tDCS cause, as well as ensuing neural effects; i.e., markers of the tDCS electric current, and the physiological state of the brain over time. Only one of these markers can be measured by existing imaging techniques, including BOLD fMRI, ASL, EEG, MEG (Polania et al., 2011; Krishnamurthy et al., 2015; Heinrichs-Graham et al., 2017; Donaldson et al., 2019) (all of which are sensitive to neurophysiological changes over time), NEMO [Neuroelectromagnetic oscillations (Bodurka and Bandettini, 2002; Chai et al., 2017; Truong et al., 2019)] and Magnetic Resonance Electrical Impedance Tomography (MREIT). The proposed technique uses typical stimulation parameters of tDCS with a DE-EPI sequence to simultaneously map (1) changes in magnetic fields along Bz (by encoding these in the phase of the MRI signal); and (2) the BOLD-contrast (by encoding these in the magnitude of the MRI signal at the 2^nd^ echo).

The tDCS current-induced magnetic field measurements were first tested in a phantom, and were observed to be accurate, reliable and sensitive to field changes on the order of 1 nT (Figure 2). It should be noted that the use of simulations as the gold standard is justified here because of the minimal assumptions involved in its calculation. Due to the phantom’s special design, accurate simulations required only (i) a simple segmentation (single-compartment, threshold-based), (ii) the specification of magnetic permeability [stable on the order of ∼ppm (Schenck, 1996)], and (iii) no assumption of electrical conductivities. Finally, comparisons between simulations and ‘Sham’ condition showed a practically negligible relationship (r_Pearson_ = −0.01, slope = 0.00, and intercept = 0.02), demonstrating the specificity of the proposed technique.

Following the phantom experiment, *in vivo* measurements were performed using a typical tDCS montage in two experiments, with the second experiment employing a test–retest paradigm. The detected current-induced magnetic fields were robust, with a mean ICC of 0.80 between test and retest measurements. Comparisons with simulations revealed a slightly weaker mean correlation, and a systematic underestimation of the induced magnetic fields by computational modeling (as shown in Figures 4, 5). This systematically higher magnetic field by *in vivo* measurements compared to simulations is consistent with previous studies (Goksu et al., 2018) and could be due to the following reasons. One factor could be model complexity. At present, simulations generally consider a 5-compartment model of the head that ignores cerebrospinal fluid (CSF), blood flow and different skull layers, which may be too simple (Opitz et al., 2015). Secondly, simulations require accurate estimates of the magnetic permeability and electrical conductivity for each tissue compartment. While the former is quite stable [varies on the order of ∼ppm across tissues (Schenck, 1996)], the latter is more variable, and has been shown to affect electric field values by up to 80% (Santos et al., 2016). At present, our simulation models utilize average conductivity values reported in literature (Gabriel et al., 1996; Wagner et al., 2004, 2014), following standard practices in the field. Our results indicate that tDCS induced electromagnetic fields are stronger than those predicted by computational modeling, which is consistent with other studies (Goksu et al., 2018).

Previous studies have shown significant changes in the phase signal of about 1 degree resulting from the BOLD-response due to finger-tapping (Feng et al., 2009; Chen and Calhoun, 2010). These changes were short-range (limited to the motor cortex), and of a bipolar pattern, with a magnetic field increase in the anterior regions and a corresponding decrease in the posterior region. No such short-range features were observed in our current-induced magnetic field data, where the induced fields range from 1 to 3 nT (i.e., 0.39–1.19 degree) *in vivo*. This could be because unlike the finger-tapping study, data in the present study was acquired during resting state. Nevertheless, this is an important consideration for future studies seeking to integrate task-fMRI together with current mapping.

### BOLD Contrast Measurement

In the proposed technique, BOLD-contrast was encoded in the magnitude of the MRI signal at the 2^nd^ echo. The magnitude of the MRI signal is sensitive to field-inhomogeneities, which can result from changes in blood oxygenation arising from neuronal activities (i.e., BOLD), or tDCS current-induced magnetic field changes (which may cause confounds).

Consequently, we first investigated tDCS current induced confounds in the phantom. Here, significant changes in the magnitude signal were observed near the wire, with the sign of the changes flipping with the direction of the applied current. The largest changes were < 5%/mA of applied current and were within 1.5 cm of the wire (where the current density was 50.93 A/m^2^). Overall, these observations suggest that significant tDCS-induced confounds in the BOLD signal can be induced near the wire. Additionally, because the size of these confounds is dependent on the current density, the confounds are also expected to reduce as the tDCS current diffuses into the brain parenchyma through the electrodes, scalp and skull. These results are consistent with (Antal et al., 2014), wherein tDCS-induced BOLD confounds were measured in cadavers, and were observed to be largely limited to the CSF and scalp.

Our group ICA analyses revealed typical large-scale RSNs, and two regional components underneath each tDCS electrode respectively (‘Cathodal’ and ‘Anodal’). These regions and networks were found to be robust between test and retest measurements (average DICE coefficient = 0.74, Figure 7), as well as across subject cohorts of Experiments 1 and 2 (average DICE coefficient = 0.55). No significant changes were observed with the ‘Anodal’ ICA component, contrary to expectations. As suggested by Antal et al. (2011), the lack of BOLD signal changes with tDCS could be due to the different physiological mechanisms between Motor-Evoked Potentials and BOLD signal. The former reflects trans-synaptic excitability changes in pyramidal neurons, and has been used to demonstrate the well-known polarity dependent shifts in cortical excitability associated with tDCS (Nitsche and Paulus, 2000; Jamil et al., 2017). The BOLD signal is primarily sensitive to changes in synaptic activity over all neurons. Nevertheless, ‘cathodal’s BOLD signal was found to significantly change at the group level. This finding is consistent with the inhibitory nature of the cathode electrode observed in electrophysiological studies (Nitsche and Paulus, 2000; Jamil et al., 2017). We further replicated our findings in the retest dataset, with a significant BOLD-signal change of similar effect size with applied current detected in the ‘cathodal’ region (and no change in ‘anodal’ region).

Finally, we investigated the possibility that the results in the gray-matter dominated cathodal region were affected by potential confounds. As described earlier, the BOLD signal is encoded in the magnitude of the MRI signal, which is sensitive to inhomogeneities in the local magnetic field. While inhomogeneities in the magnetic field can arise from changes in blood oxygenation arisen from neuronal activities (i.e., BOLD), they can also result from tDCS current-induced magnetic field changes, causing potential confounds. To address this concern, estimates of the current-induced magnetic field inhomogeneities were calculated from the measured current-induced magnetic fields, and the predicted confounds were an order of magnitude weaker than the observed effects (Table 1). Overall, our results suggest that the detected decreased BOLD signal in the ‘Cathodal’ region arises from changes in blood oxygenation associated with neuronal activities. Future research will expand the method used in our simulations and (Jog, 2017) to develop a general framework that can estimate potential contamination of the BOLD-contrast for a given experimental design. To the best of our knowledge, such a framework does not exist. This work will contribute to planning future tDCS studies with BOLD fMRI, as well as interpreting existing ones (Antal et al., 2011; Krishnamurthy et al., 2015; Alekseichuk et al., 2016).

Note that since both the magnetic field and BOLD data are acquired concurrently with the applied current switched on/off in a block design, we expect the two to be correlated to the block design (and likely to each other). However, there may be subtle differences between the two timeseries as a result of (a) the magnitude of response: the current induced magnetic fields are linearly proportional to the applied current (Ampere’s law), while the BOLD changes will depend on the local neuronal response, and (b) the shape of the response: the BOLD response is the underlying neuronal response (which, if significant, is correlated with the applied block design) convolved with the hemodynamic response function. The current induced magnetic field on the other hand, is linearly proportional to the applied block design.

### Limitations and Future Directions

The proposed current mapping approach uses MRI to detect tDCS current-induced magnetic field changes along the static magnetic field (Bz). In the absence of additional information, all three components of the magnetic field need to be measured to reconstruct the underlying current density. One way to address this limitation would be to measure subjects in at least three different orientations (Joy et al., 1989). An alternative approach that does not require additional measurements, uses the measured component of the induced field to fit electromagnetic model parameters (Dutta and Dutta, 2013). This approach is suggested by the following three observations: (a) the measured current-induced magnetic fields (along Bz) are well-correlated with simulations (Figures 4, 5), (b) unlike *in vivo* measurements, simulations can predict the undetected components of the magnetic field and current density, and (c) the parameter space of the simulated models is not large. Tissue electrical conductivities would be a good starting point for optimization based on the following factors: they have been shown to be variable across subjects; they can affect electric field values by up to 80% in the brain; and current models utilize average conductivity values reported in literature (Gabriel et al., 1996; Wagner et al., 2004; Santos et al., 2016).

Recently, Chen et al. (2018, 2019) have applied group information guided ICA (GIG-ICA) to reveal phase-based resting state networks in a cohort of 600 subjects. The networks were identified using Pearson correlation coefficient, and these correlated signals could be a potential source of confound in the tDCS-current induced magnetic field measurements, if correlated with the utilized block design. Addressing the disentanglement of phase-based resting state network signals from the tDCS current induced magnetic field signal (possibly by comparing the size of the induced phase changes) will be the focus of our future work.

Another potential future direction of research could be the utilization of short duration currents. Though not applicable to tDCS, such a paradigm would be consistent with transcranial alternating current stimulation (tACS). tACS in resting brain along with a simple motor task (e.g., finger tapping) can potentially be used for measuring current-induced fields as well as associated BOLD signal changes, analysis of which could potentially provide insights into the mechanisms underlying tACS.

In this study, concurrent tDCS-BOLD measurements revealed typical resting state brain networks. These networks were robust, and could be used to track brain states [through decoding of connectivity patterns (Shirer et al., 2012; Chang et al., 2016)] as well as state-transitions associated with tDCS current. In addition to detecting RSNs, the proposed technique also revealed local changes in neuronal activity with the applied current at the group level (Figures 6, 7, normalized BOLD-signal change/mA tDCS). While a simple *t*-test was used to examine effects at the group level, the individual estimates of the BOLD signal change per mA tDCS applied could be investigated for correlations with behavioral metrics or clinical outcome measures, although the latter metrics were not collected in this proof-of-concept study. Nevertheless, these concurrent measurements allow investigation of the relationship between the tDCS currents (as measured by the magnetic field), the response of the brain to tDCS (measured by BOLD-contrast), and clinical outcomes. Unveiling the relationships between these factors could help understand the mechanisms of tDCS, and provide insights into resolving the inter-subject variability observed in the clinical translation of tDCS.

## Conclusion

In this work we present a novel technique that provides the capability to *simultaneously* map (i) the tDCS current-induced magnetic fields (a direct marker of the tDCS current), and (ii) BOLD-contrast (a marker of neurophysiological changes), with full brain coverage. With further refinement, this technique may allow the visualization of target engagement, in parallel with a means to study the response of the brain to tDCS at the group level. Our technique also contributes to addressing confounding factors in applying BOLD fMRI concurrently with tDCS.

## Data Availability Statement

The data is available on the NIMH Data Archive (https://nda.nih.gov/). All data, codes and workflow associated with this study will be made available from the corresponding author upon request.

## Ethics Statement

The studies involving human participants were reviewed and approved by the Institutional Review Board of the University of Southern California and the Institutional Review Board of the University of California, Los Angeles. The patients/participants provided their written informed consent to participate in this study.

## Author Contributions

MJ, KJ, LY, and DW designed the overall study. LY performed the MRI sequence development, with inputs from MJ. MJ and KJ performed the data acquisition. MJ, KJ, KN, and DW analyzed the data. LP, KN, MB, and DW provided funding. All authors wrote and revised the manuscript.

## Conflict of Interest

MB and LP are co-inventors for patents on transcranial electric stimulation held by the City University of New York and hold shares in Soterix Medical, Inc. The remaining authors declare that the research was conducted in the absence of any commercial or financial relationships that could be construed as a potential conflict of interest.
