# Supplementary material for: Concurrent Imaging of Markers of Current Flow and Neurophysiological Changes During tDCS
Source: Front Neurosci. 2020 Apr 21;14:374. doi: 10.3389/fnins.2020.00374 (PMC7186453; doi:10.3389/fnins.2020.00374)
Supplement: Supplementary file 1 [file Data_Sheet_1.docx]

Supplementary Material

**Supplementary S.1: Order of tDCS currents applied during scans**

As described in methods, the first protocol was developed on a Siemens 3T Trio scanner with a comparatively older software version and a slower scan rate. Here, each DE-EPI scan consisted of 4 different current-values (0, 0.5, 1 and 1.5 mA) applied in blocks of 3 minutes each (shown in Supplementary Fig. 2.A). After the scanner upgrade, an improved protocol with better statistical power was developed. Here, each scan consisted of 4 values of tDCS currents (0, 1, 1.5 and 2mA) applied in a fixed pseudo-random order in a block design, as shown in Supplementary Fig. 2.B. Ramp times between blocks were 30 sec. for both protocols.


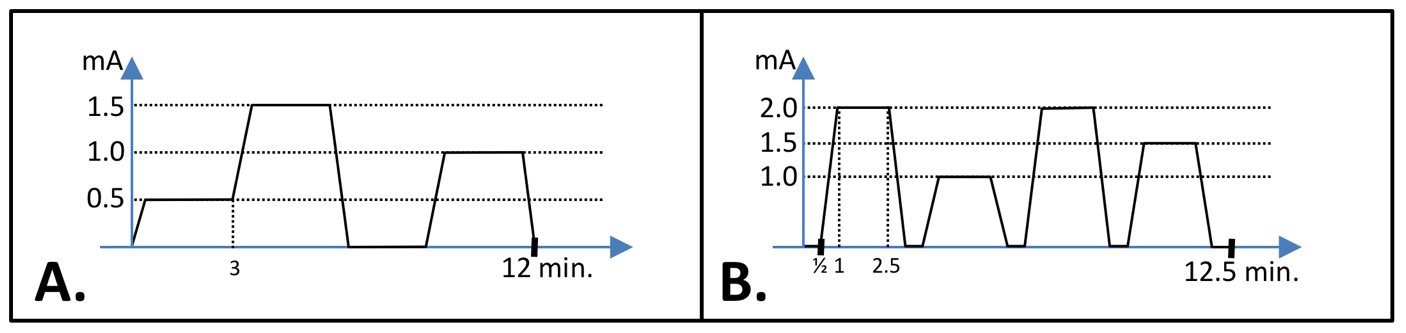

 **Supplementary Fig. S1.**

**Supplementary S.2**: **BOLD-signal confounds**

Assuming that the magnetic field changes by $\Delta B_{z}$ over a voxel, the worst-case inhomogeneity is represented by the case wherein half the spins experience $\Delta B_{z}$ (and the rest experience 0). In this case, the observed signal ($M_{Obs}$)

$$M_{Obs}=\frac{m}{2}.cos\left( \frac{\phi}{2} \right)+\frac{m}{2}.cos(-\frac{\phi}{2})$$

$$\phi=2\pi.\gamma.\Delta B_{z}.TE$$

Here, m = total magnetization signal in the voxel,and the rest of the constants are as defined in Equation [1] in the main manuscript. Thus, the fractional signal change
in this model (Model#1 in the manuscript) is, fs

$$fs=\frac{m-M_{Obs}}{m}=1-cos\left( \frac{\phi}{2} \right)$$

Relaxing the worst-case, we assume that the magnetic field changes by $\Delta B_{z}$ linearly over a voxel, i.e. [0,L], where L denotes the size of the voxel. Here,

$$M_{Obs}=\int_{-\frac{\phi}{2}}^{+\frac{\phi}{2}} \frac{m}{L}.cos\left( \theta\right).dx$$

$$\frac{x}{L}=\frac{\theta}{\phi}$$

Solving the equation,

$$M_{Obs}=m.\frac{\sin\left( \frac{\phi}{2} \right)}{\frac{\phi}{2}}$$

and

$$fs=\frac{\sin\left( \frac{\phi}{2} \right)}{\frac{\phi}{2}}$$

is the fractional change in signal in Model #2.

**Supplementary S.3**: **Rationale behind designating regions as ‘Cathodal and ‘Anodal’**

Here, we show that ‘Cathodal’ and ‘Anodal’ regions identified by the group ICA are indeed underneath the cathode and anode electrode sites.

Supplementary Table S3 (below) shows the peak coordinates of ‘Cathodal’ and ‘Anodal’ regions in each dataset. All coordinates are in mm, and in MNI space. Additionally, we calculated the distance of each peak from cortical projections of C3 and C4; the latter being the locations of cathode and anode electrodes respectively. As can be seen, the distances are well within the dimensions of the electrodes used (=70x50 mm, centered at C3/C4), and it can be concluded that the peak-coordinates are underneath the electrodes.

Note that the cortical projections of C3 and C4 were obtained from :
Okamoto M, Dan H, Sakamoto K, Takeo K, Shimizu K, Kohno S, et al. Three-dimensional probabilistic anatomical cranio-cerebral correlation via the international 10-20 system oriented for transcranial functional brain mapping. NeuroImage. 2004;21(1):99-111


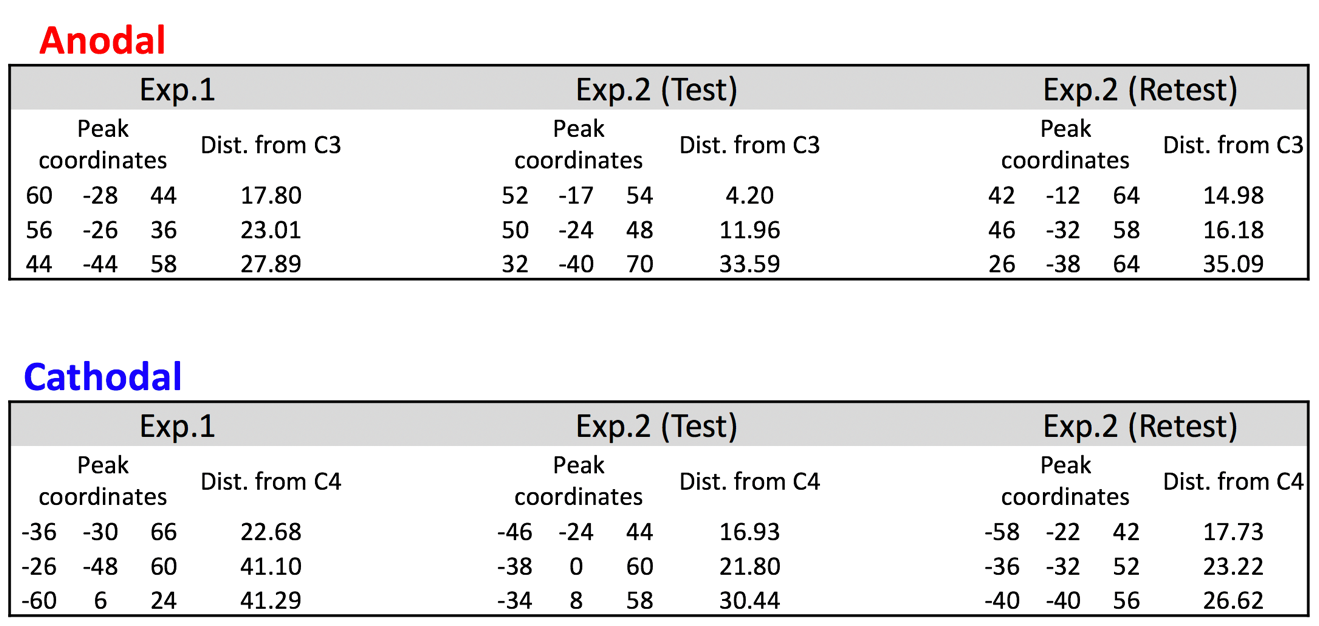


**Supplementary Table S2.**
